# Exposure to PBDEs in the Office Environment: Evaluating the Relationships Between Dust, Handwipes, and Serum

**DOI:** 10.1289/ehp.1003271

**Published:** 2011-06-30

**Authors:** Deborah J. Watkins, Michael D. McClean, Alicia J. Fraser, Janice Weinberg, Heather M. Stapleton, Andreas Sjödin, Thomas F. Webster

**Affiliations:** 1 Department of Environmental Health, and; 2 Department of Biostatistics, Boston University School of Public Health, Boston, Massachusetts, USA; 3 Nicholas School of the Environment, Duke University, Durham, North Carolina, USA; 4 Division for Laboratory Sciences, National Center for Environmental Health, Centers for Disease Control and Prevention, Atlanta, Georgia, USA

**Keywords:** dust, flame retardants, handwipes, indoor exposure, office, PBDEs

## Abstract

Background: Polybrominated diphenyl ethers (PBDEs) have been widely used as flame retardants in consumer products and are ubiquitous in residential indoor air and dust. However, little is known about exposure in the office environment.

Objectives: We examined relationships between PBDE concentrations in the office environment and internal exposure using concurrent measurements of PBDEs in serum, handwipes, and office dust.

Methods: We collected serum, dust, and handwipe samples from 31 participants who spent at least 20 hr/week in an office. We used a questionnaire to collect information about work and personal habits.

Results: We found positive associations between PBDEs in room dust, handwipes (a measure of personal exposure), and serum. PBDE office dust concentrations were weakly correlated with measurements in handwipes: *r* = 0.35 (*p* = 0.06) for pentaBDE (sum of BDE congeners 28/33, 47, 99, 100, and 153) and 0.33 (*p* = 0.07) for BDE-209. Hand washing also predicted pentaBDE levels in handwipes: low hand-washers had 3.3 times the pentaBDE levels in their handwipes than did high hand-washers (*p* = 0.02). PentaBDE in handwipes predicted pentaBDE levels in serum (*p* = 0.03): Serum concentrations in the highest handwipe tertile were on average 3.5 times the lowest handwipe tertile. The geometric mean concentration of pentaBDEs in serum was 27 ng/g lipid. We detected BDE-209 in 20% of serum samples, at levels ranging from < 4.8 to 9.7 ng/g lipid.

Conclusion: Our research suggests that exposure to pentaBDE in the office environment contributes to pentaBDE body burden, with exposure likely linked to PBDE residues on hands. In addition, hand washing may decrease exposure to PBDEs.

Polybrominated diphenyl ethers (PBDEs) are brominated flame retardants that have been widely used over the past few decades. PBDEs were originally manufactured in three commercial formulations: penta, octa, and deca. The penta formulation was mainly used in North America as an additive to polyurethane foam in furniture and carpet padding, whereas the octa and deca formulations were added to plastic polymers used in electronics. Animal studies indicate that PBDE exposure affects neurodevelopment and disrupts the endocrine system (Birnbaum and Staskal 2004). Epidemiologic studies have linked exposure to the penta congeners to changes in thyroid hormone homeostasis (Meeker et al. 2009), fertility (Akutsu et al. 2008; Harley et al. 2010), male reproductive hormones (Meeker et al. 2009), and neurodevelopment (Herbstman et al. 2010; Roze et al. 2009), as well as cryptorchidism (Main et al. 2007) and decreased birth weight and length (Chao et al. 2007).

In 2004, the penta and octa formulations were voluntarily withdrawn from the U.S. market by manufacturers and banned in the European Union. Deca is currently being phased out in the European Union, and manufacturers recently announced plans to phase out deca in the United States by the end of 2013 [U.S. Environmental Protection Agency (EPA) 2010]. Nevertheless, many products are still in use that were manufactured or sold before these changes. penta and octa were recently added to the Stockholm Convention and recognized as persistent organic pollutants (Stockholm Convention 2010). Because of slow turnover of products containing PBDEs and the long half-life of PBDEs in the environment, human exposure to these compounds will continue for many years.

As a result of widespread use in consumer products and the persistence of these compounds, PBDEs are ubiquitous in residential indoor air and dust (Allen et al. 2007, 2008), and bioaccumulate in human tissues such as serum and breast milk (Frederiksen et al. 2009). PBDE body burdens have been found to be associated with house dust (Frederiksen et al. 2010; Johnson et al. 2010; Wu et al. 2007) and diet (Fraser et al. 2009), but primary exposure pathways are still uncertain. Suspected routes of exposure to PBDEs in the indoor environment include incidental dust ingestion, dermal exposure, and inhalation (Allen et al. 2007; Stapleton et al. 2005). PBDEs can be detected in handwipes, suggesting that a key exposure route may be incidental ingestion during eating, biting nails, or other hand-to-mouth behaviors (Stapleton et al. 2008).

Because previous U.S. studies of PBDEs have focused on residential exposures, little is known about exposure in offices. Office workers are often in close proximity to furniture, computers, and other equipment that may contain flame retardants. Using chamber experiments, Destaillats et al. (2008) demonstrated that some personal computers emit PBDEs and other semivolatile organic compounds, but it is unclear how this translates to human exposure. Zhang et al. (2009) developed a fugacity model for an office to estimate emission rates of PBDEs from specific office products. However, because only one office was sampled, uncertainties remain regarding the generalizability of these estimates. Because biological measures of exposure were not collected, the authors were unable to connect PBDEs in the office to body burden.

Office environments are often considered public space, where furniture may be required to meet strict fire codes. For example, the city of Boston requires that office furniture (but not residential furniture) meet California fire retardant standards (Boston Fire Department 1995). Such fire codes may lead to increased exposure to PBDEs and other flame retardants (Zota et al. 2008).

We hypothesize that an abundance of office equipment and increased use of flame retardants in office furniture may increase exposure to PBDEs. The goals of the present study were to examine relationships between PBDE concentrations in offices and internal exposure using concurrent measurements of PBDEs in serum, handwipes, and office dust.

## Materials and Methods

*Study design.* We recruited a convenience sample of 31 adults who work and live in the Boston, Massachusetts (USA), area by posting announcements and flyers in multiple office buildings, as well as through word of mouth. The study population consisted of 26 females and 5 males, was 90% white, and had a median age of 49 years. To be eligible for participation, subjects had to work at least 20 hr a week in an office (excluding cubicles) and be a healthy nonsmoker. Offices were located in eight different buildings. In shared offices, only one person was allowed to participate. We conducted the field effort from January through March 2009. Active participation for each subject lasted 1 week and included the collection of an office dust sample, handwipe sample, blood sample, and questionnaire data from each participant. The Boston University Medical Center institutional review board approved the study protocol, and all participants gave their informed consent.

*Dust samples.* Investigators collected dust samples into cellulose extraction thimbles (Whatman Inc., Piscataway, NJ) as previously described (Allen et al. 2008). Each office was vacuumed for approximately 10 min, capturing dust from the entire floor surface area of the room, including accessible floor space under desks and the tops of immovable furniture. After sample collection, thimbles were wrapped in aluminum foil, sealed in polyurethane bags, and stored at room temperature until processed. We collected dust field blanks by vacuuming sodium sulfate powder (as a surrogate for dust) from a clean aluminum foil surface. Dust samples were sieved to collect particles < 500 μm in size, placed in clean amber glass jars, and stored at –20°C. We analyzed dust samples for 37 PBDE congeners using gas chromatography/mass spectrometry operated in electron-capture negative-ionization mode (GC/ECNI-MS), as previously described (Stapleton et al. 2008).

*Handwipe samples.* We collected handwipe samples from participants at their office environment, typically in the afternoon and at least 60 min after their last hand washing. We immersed a 3 × 3 inch sterile gauze pad in 3 mL isopropyl alcohol and then wiped the palm and back of the hand from wrist to fingertips. The handwipe was then placed in a clean glass vial, wrapped in foil and bubble wrap, and stored at –20°C. Left and right hands were sampled separately but extracted and analyzed together, providing one measurement per participant. We paired a field blank wipe sample with the collection of each handwipe by soaking a gauze pad in isopropyl alcohol and placing it directly into the glass vial. Handwipe samples were analyzed for 37 PBDE congeners using GC/ECNI-MS, using methods described previously (Stapleton et al. 2008).

We normalized PBDE measurements to hand surface area (nanograms per square centimeter), calculated as described in the U.S. EPA *Exposure Factors Handbook* (U.S. EPA 1997). Normalized PBDE concentrations were highly correlated with the mass of PBDEs in handwipes (data not shown). Because of this high correlation and the uncertainty associated with hand surface area calculation, we used the PBDE mass in handwipes for all data analysis.

*Blood samples.* A phlebotomist collected one 10-mL red-top Vacutainer tube of blood from each participant at the end of the work week. Tubes were allowed to coagulate at room temperature for 1–2 hr and centrifuged for 15 min at 1,000 × *g*. Serum from each individual was separated into aliquots for PBDE and lipid analysis and stored at –20°C in 10-mL amber glass vials and 2-mL polypropylene vials, respectively. Serum samples were analyzed for lipids and 11 PBDE congeners at the Centers for Disease Control and Prevention using established methods (Sjödin et al. 2004).

*Questionnaire.* We designed and administered a questionnaire to collect information about work and personal habits, including the average numbers of hours per week spent in their offices and the average numbers of times per day the participants washed their hands, as well as personal characteristics such as sex, height, and weight. Information on hand washing was collected as < 2 times/day, 2–4 times/day, 4–6 times/day, or > 6 times/day. For data analysis we collapsed the four categories into two: < 4 times/day (low hand-washers) and ≥ 4 times/day (high hand-washers). At the time of handwipe collection, we asked participants how long it had been since they had last washed their hands. We also measured and recorded surface area, temperature, relative humidity, and other characteristics of the office.

*Data analyses.* We blank-corrected samples on a congener-specific basis. We corrected dust samples by subtracting the mean of the dust field blanks, and handwipes by subtracting each individual’s paired wipe field blank. Serum samples were corrected using the mean of within-run laboratory blanks. Limits of detection (LODs) for dust and handwipe samples were determined as three times the standard deviation of the appropriate blanks. We used the laboratory instrument detection limit as the LOD when congeners were not detected in the field blanks or there were insufficient data to calculate an LOD. For all samples, concentrations < LOD were substituted with a value of LOD/2.

PBDE data appeared log-normally distributed, and Shapiro-Wilk tests indicated the data were nonnormal. Because tests of natural log-transformed data indicated they were consistent with normality, we transformed data before analysis when appropriate. We used Spearman correlations to determine associations between continuous variables while minimizing the influence of outliers. We used linear regression models to determine predictors of ln PBDE concentrations in serum and handwipes. To aid interpretation of model results ®-coefficient estimates were exponentiated (*e*^®^), producing the multiplicative change in outcome. Because of low detection, logistic regression was used to determine predictors of BDE-209 detection in serum (detect vs. nondetect). To minimize the effect of skewed data and outliers, we created categorical variables from continuous dust and handwipe data: Two-level variables (low, high) were created using the median as a cut-point; three-level variables (low, medium, high) were created using tertiles. All statistical analyses were performed using SAS (version 9.1; SAS Institute Inc., Cary, NC), with statistical significance defined as 〈 = 0.05.

## Results

*Office dust.* Table 1 presents summary statistics for PBDE concentrations in office dust. pentaBDE concentrations ranged from 141 to 61,264 ng/g dust, with a geometric mean (GM) of 2,167 ng/g. We define pentaBDE here as the sum of BDE congeners 28/33, 47, 99, 100, and 153, the congeners detected in > 50% of samples in all three media: dust, handwipes, and serum. For example, we did not include BDE-154 because we detected it in only 30% of serum samples. We detected individual penta congeners in 84–100% of dust samples. We detected BDE-183, the main component of octa, and BDE-209, the main component of deca, in 100% of dust samples, with GMs of 81 ng/g and 4,204 ng/g, respectively. Individual PBDE congeners measured in office dust were correlated with one another in a pattern generally representing the three commercial PBDE mixtures: penta, octa, and deca [see Supplemental Material, “Spearman Correlations within Sample Type,” Table 1 (http://dx.doi.org/10.1289/ehp.1003271)].

**Table 1:** Measurements of PBDEs in office dust, handwipes, and serum (*n* = 31).

Office dust (ng/g)					Handwipes (ng)					Serum (ng/g lipid)				
BDE congener	Percent detection	GM (GSD)	Range	Percent detection	GM (GSD)	Range	Percent detection	GM (GSD)	Range
Penta																		
BDE-28/33		87		7.5 (4.9)		< 0.4 to 207		90		0.6 (3.4)		< 0.1 to 5.3		80		1.1 (2.6)		< 0.5 to 4.4
BDE-47		100		697 (3.7)		36.8 to 19,494		100		32.8 (3.5)		5.7 to 1,053		97		14.2 (3.0)		< 2.1 to 178
BDE-49		87		18.8 (8.4)		< 0.4 to 612		87		0.7 (5.6)		< 0.05 to 13.6						
BDE-66		84		9.0 (10.5)		< 0.2 to 504		94		0.6 (4.2)		< 0.05 to 11.0		7		NC		< 0.5 to 1.0
BDE-75		87		39.8 (6.1)		< 0.4 to 227		81		0.5 (3.0)		< 0.2 to 5.0						
BDE-85/155		97		49.6 (5.6)		< 0.2 to 3,085		100		1.0 (4.0)		0.2 to 59.1		33		NC		< 0.5 to 4.3
BDE-99		97		915 (6.1)		< 0.4 to 32,831		100		27.4 (3.9)		4.4 to 1,428		60		2.5 (3.0)		< 1.9 to 45.9
BDE-100		100		195 (4.3)		12.7 to 8,672		100		5.3 (3.9)		0.9 to 260		94		2.7 (3.4)		< 0.5 to 51.4
BDE-138		100		17.9 (4.9)		1.6 to 958		81		0.2 (4.4)		< 0.05 to 13.0						
BDE-153		100		138 (4.9)		11.1 to 5,973		100		1.9 (3.8)		0.3 to 118		97		5.0 (3.7)		< 0.5 to 173
BDE-154		100		115 (4.5)		7.6 to 5,202		100		1.7 (3.8)		0.3 to 98.5		30		NC		< 0.5 to 5.5
PentaBDE*a*				2,167 (4.3)		141 to 61,264				70 (3.7)		13.8 to 2,864				27.7 (3.0)		3.4 to 348
Octa																		
BDE-183		100		81.2 (4.0)		14.9 to 12,970		84		0.3 (3.3)		< 0.1 to 8.7		13		NC		< 0.5 to 4.7
BDE-196		100		29.1 (3.0)		6.7 to 2,858		NA										
BDE-197		100		32.4 (4.1)		4.2 to 6,109		52		0.2 (2.7)		< 0.2 to 4.5						
BDE-201		97		4.9 (3.0)		< 1.0 to 359		16		NC		< 0.2 to 0.8						
Deca																		
BDE-206		100		153 (2.7)		29.1 to 3,395		16		NC		< 0.2 to 3.0						
BDE-207		100		125 (3.0)		21.9 to 4,312		58		0.2 (4.0)		< 0.1 to 2.1						
BDE-208		100		61.8 (2.9)		10.4 to 1,710		52		0.1 (3.0)		< 0.1 to 0.9						
BDE-209*b*		100		4,204 (2.9)		912 to 106,204		94		11.8 (3.2)		< 1.0 to 105		20		NC		< 4.8 to 9.7
GSD, geometric standard deviation; NA, not available; NC, not calculated because of detection in < 50% of samples. Congeners with < 50% detection in both office dust and handwipes are not reported (BDE congeners 17, 25, 30, 71, 116, 119, 156, 171, 176, 179, 181, 184, 188, 190, 191, 202, and 205). **a**PentaBDE comprises BDE congeners 28/33, 47, 99, 100, and 153 (congeners detected in > 50% of samples within all three media: dust, handwipes, and serum). **b**BDE-209 range was ≤ 28 to 50 pg/g serum, not adjusted for lipid.																		

*Handwipes.* PentaBDE measurements in handwipes ranged from 14 to 2,864 ng, with a GM of 70 ng (Table 1). We detected most individual penta congeners in 87–100% of handwipe samples. We detected BDE-183 in 84% of handwipes, with a GM of 0.3 ng, whereas we detected BDE-209 in 94% of handwipe samples, with a GM of 12 ng. Individual PBDE congeners in handwipes were also correlated in a pattern reflecting the three commercial mixtures [see Supplemental Material, “Spearman Correlations within Sample Type,” Table 2 (http://dx.doi.org/10.1289/ehp.1003271)].

**Table 2:** Predictors of PBDEs in handwipes collected in the office environment.

PentaBDE							BDE-183							BDE-209						
Crude*a*			Adjusted*b*			Crude*a*			Adjusted*b*			Crude*a*			Adjusted*b*		
Predictor	β-Coefficient (SE)	*p*-Value	β-Coefficient (SE)	*p*-Value	β-Coefficient (SE)	*p*-Value	β-Coefficient (SE)	*p*-Value	β-Coefficient (SE)	*p*-Value	β-Coefficient (SE)	*p*-Value
Office dust																								
Low		Reference				Reference				Reference				Reference				Reference				Reference		
High		0.89 (0.45)		0.06		0.77 (0.43)		0.08		0.67 (0.41)		0.12		0.64 (0.42)		0.14		0.22 (0.42)		0.61		0.19 (0.45)		0.67
Hand washings/day																								
≥ 4		Reference				Reference				Reference				Reference				Reference				Reference		
< 4		1.19 (0.50)		0.02		1.08 (0.49)		0.03		0.43 (0.49)		0.39		0.33 (0.48)		0.49		0.16 (0.48)		0.74		0.10 (0.51)		0.85
Sex																								
Female		Reference				NA				Reference				NA				Reference				NA		
Male		0.81 (0.63)		0.21						–0.004 (0.59)		0.99						–0.65 (0.56)		0.26				
Age (years)		0.02 (0.02)		0.31		NA				0.01 (0.02)		0.72		NA				–0.02 (0.02)		0.16		NA		
Time since hand washing*c*		0.004 (0.003)		0.18		NA				0.004 (0.003)		0.11		NA				0.003 (0.003)		0.21		NA		
BMI		–0.07 (0.06)		0.19		NA				0.02 (0.05)		0.70		NA				–0.02 (0.05)		0.63		NA		
NA, sex, age, time since hand washing, and BMI were not included in the adjusted models. SE, standard error. β-Coefficients represent the change in the natural log of the PBDE mass measured on handwipes relative to the reference group for categorical variables, or per unit change for independent continuous variables (age, BMI, time since hand washing). **a**Crude models include only a single independent variable. **b**Adjusted models include dichotomous office dust variable (low, high) and dichotomous hand-washing variable (≥ 4 and < 4 times/day). **c**Time, in minutes, between handwipe sample collection and last hand washing.																								

*Dust and handwipe associations.* PentaBDE measurements in handwipes were weakly correlated with pentaBDE concentrations in office dust, with a Spearman correlation coefficient of 0.35 [*p* = 0.06; see Supplemental Material, Figure 1 (http://dx.doi.org/10.1289/ehp.1003271)]. We found similar associations for BDE-183 (*r* = 0.47, *p* = 0.008) and BDE-209 (*r* = 0.33, *p* = 0.07). We detected other octa and deca congeners in < 60% of dust and handwipe samples, so we did not include them in data analyses.

**Figure 1:**
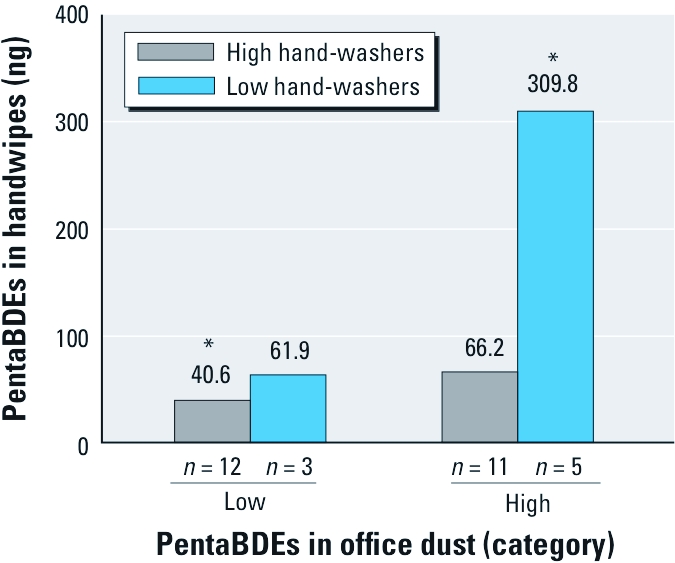
GM of PentaBDE measurements in handwipes by office dust and hand-washing categories. *Significant difference between low-dust/high-wash and high-dust/low-wash groups (*p* < 0.05).

To further examine associations between office dust and handwipes, we divided office dust concentrations of pentaBDEs, BDE-183 and BDE-209 into two categories (low, high). We used linear regression to determine if the categorical office dust variables were a significant predictor of continuous BDE measurements in handwipes. Table 2 presents the parameter estimates, SEs, and *p*-values for these models, with the ®-coefficient estimate representing the mean difference in handwipe PBDEs on the natural log scale. The exponentiated ®-coefficient estimate represents the multiplicative change in outcome. For example, the ®-coefficient for pentaBDE (sum of BDE congeners 28/33, 47, 99, 100, and 153) was 0.89, meaning that those in the high-office-dust category had on average *e*^0.89^, or 2.4 times, the mass of pentaBDE in their handwipes compared with those in the low-office-dust category (*p* = 0.06). BDE-183 and -209 measurements in handwipes were not significantly associated with office dust category (Table 2).

*Hand washing.* Table 2 shows that participants who washed their hands fewer than four times per day had on average 3.3 times (*e*^1.19^) the mass of pentaBDE in their handwipes compared with those who washed their hands four or more times per day (*p* = 0.02). When we added the dichotomous office dust and hand-washing variables to the model as predictors of pentaBDE levels in handwipes, ®-coefficient estimates changed only minimally (Table 2). BDE-209 and BDE-183 measurements in handwipes were not associated with hand-washing category.

We next added to the model an interaction term consisting of the dichotomous hand-washing variable and the dichotomous office dust variable to determine if the relationship between pentaBDE in office dust and handwipes depended on how often participants reported they washed their hands. Although the interaction term was not statistically significant (*p* = 0.26), participants with high concentrations of pentaBDE in their office dust who washed their hands fewer than four times per day had much higher pentaBDE handwipe measurements (GM = 310 ng) than did participants who had high concentrations in their office dust but washed their hands at least four times per day (GM = 66 ng; Figure 1).

Time between handwipe sample collection and last hand washing, and sex, age, and body mass index (BMI) were not significant predictors of PBDEs in handwipes (Table 2), and associations between office dust and handwipes did not substantially change when we added these variables to the models (data not shown).

*Serum.* Table 1 presents summary statistics for PBDE concentrations in serum. PentaBDE concentrations ranged from 3.4 to 348 ng/g lipid, with a GM of 28 ng/g lipid. We detected BDE-183 in 13% of serum samples, ranging from < LOD (0.5 ng/g lipid) to 4.7 ng/g lipid. We detected BDE-209 in 20% of serum samples, ranging from < LOD (4.8 ng/g lipid) to 9.7 ng/g lipid (< 28 to 50 pg/g serum).

Serum concentrations of individual pentaBDE congeners, including BDE-153, were all highly correlated with one another and with the summed pentaBDE measure [see Supplemental Material, Table 3 (http://dx.doi.org/10.1289/ehp.1003271)]. Because we did not detect BDE-183 and BDE-209 in most serum samples, we did not estimate associations of serum with handwipes or office dust measures for these congeners.

**Table 3:** Univariate predictors of PentaBDE concentrations in serum.

Predictor	*n*	β-Coefficient (SE)	Serum GM (ng/g lipid)	*p*-Value
Handwipe category								0.03
Low		10		Reference		14.0		
Medium		11		0.80 (0.45)		31.2		0.09
High		10		1.24 (0.45)		48.6		0.01
Hand washings/day								
≥ 4		23		Reference		20.6		
< 4		8		1.10 (0.42)		62		0.01
Sex								
Female		26		Reference		24.5		
Male		5		0.73 (0.53)		50.7		0.18
Age (years)		NA		–0.01 (0.01)		NA		0.39
BMI		NA		–0.04 (0.05)		NA		0.40
NA, not applicable; SE, standard error. β-Coefficients represent the change in the natural log of PentaBDE serum concentrations relative to the reference group for categorical variables, or per unit change for independent continuous variables (age, BMI).								

**Figure 2:**
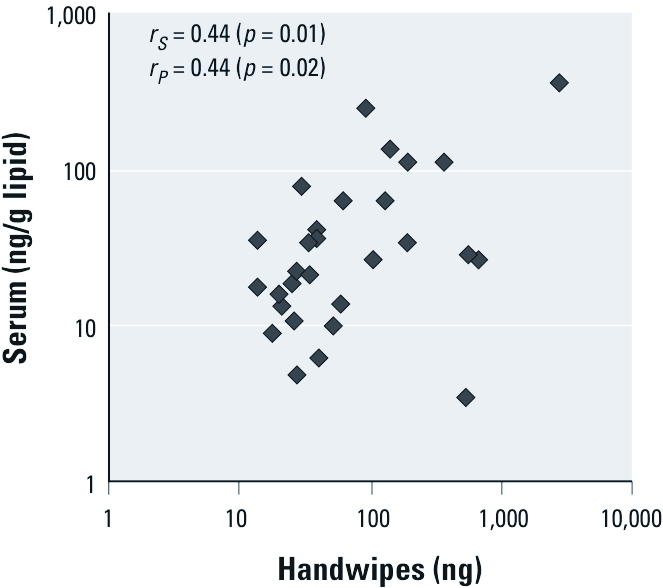
Correlation of PentaBDE in serum versus handwipes (*n* = 30): data for Pearson (*r*_P_) correlation coefficients were natural log transformed; *r*_s_, Spearman correlation coefficient.

*Handwipe and serum associations.* PentaBDE levels in handwipe and serum samples were correlated, with a Spearman correlation coefficient of 0.44 (*p* = 0.01; Figure 2). We divided pentaBDE levels in handwipes into three categories (low, medium, high) and entered them into a regression model as predictors of pentaBDE serum concentrations. Table 3 presents ®-coefficient estimates from this model expressed as mean differences in serum pentaBDE on the natural log scale. Handwipe category was a significant predictor of pentaBDE concentrations in serum (*p* = 0.03), with serum concentrations in the high-handwipe category approximately 3.5 times (*e*^1.24^) the serum concentrations in the low-handwipe category, and concentrations in the medium-handwipe category 2.2 times (*e*^0.80^) the concentrations in the low-handwipe category.

Hand-washing category (high, low) was also a significant predictor of pentaBDE concentrations in serum, with low hand-washers having on average 3.0 times (*e*^1.10^) the concentration of pentaBDE in their serum compared with high hand-washers (*p* = 0.01). Because we consider hand washing to affect handwipes, we did not include both hand washing and handwipes in the same regression models for serum.

Because we detected BDE-209 in only 20% of serum samples, we analyzed serum BDE-209 as detect versus nondetect using logistic regression. Using BDE-209 levels in handwipes as a continuous predictor produced a nonsignificant odds ratio (OR) of 1.02 (*p* = 0.14), that is, a 2% increase in the odds of detecting BDE-209 in serum per unit increase of BDE-209 in handwipes. Use of high versus low categories of handwipe BDE-209 produced a nonsignificant OR of 2.4 (*p* = 0.37), suggesting that participants with high levels of BDE-209 on their hands had, on average, 2.4 times the odds of having BDE-209 detected in their serum compared with participants with low levels on their hands. These results were not statistically significant, at least partly because of the small percentage of serum samples with detectable BDE-209. Hand-washing category was not a significant predictor of BDE-209 detection in serum (data not shown). Additional research is needed to fully characterize the relationship between BDE-209 on hands and BDE-209 in serum.

*Office dust and serum associations.* PentaBDE concentrations in office dust were not significantly correlated with serum pentaBDE (*r* = 0.22, *p* = 0.25), and we found no association between BDE-209 concentrations in office dust and detection of BDE-209 in serum (OR = 1.0, *p* = 0.66). The association between pentaBDE concentrations in office dust and serum did not change when we entered hand-washing category (high, low) into the model (data not shown). Age, sex, and BMI were not significant predictors of pentaBDE concentrations in serum (Table 3), and ®-coefficient estimates for handwipe category and the hand-washing variable did not substantially change when we added these characteristics to the models (data not shown).

## Discussion

We collected paired office dust, handwipe, and serum samples from each participant in this study, allowing us to examine associations between sample types and thereby explore PBDE exposure pathways. Some previous studies have shown associations between dust and biological measures of pentaBDE (Frederiksen et al. 2010; Johnson et al. 2010; Wu et al. 2007), whereas others have not (Fromme et al. 2009; Roosens et al. 2010). Discrepancies among studies may be due to differences in dust collection methods or geographical disparities in penta use. The present study is the first to examine the links among measurements in microenvironments, personal exposure, and internal dose [see Supplemental Material, Figure 2 (http://dx.doi.org/10.1289/ehp.1003271)]. Handwipes provide a measure of personal exposure, an intermediate step that may explain how PBDEs in dust (measured in environment) become PBDEs in people (measure of absorbed dose).

Although the correlation between concentrations of pentaBDE congeners (in this study, sum of BDE congeners 28/33, 47, 99, 100, and 153) in office dust and serum was only *r* = 0.22 (*p* = 0.25) and the association between office dust and handwipes was weak (*r* = 0.35, *p* = 0.06), the association between handwipes and serum was stronger (*r* = 0.44, *p* = 0.01). These results suggest that exposure to pentaBDE congeners in the office environment contributes to pentaBDE body burden. However, handwipes may integrate exposure across multiple microenvironments and probably do not reflect exposure solely from offices. Handwipes may also be a more biologically relevant measure of dust exposure than dust collected from an entire room.

Hand washing was also a significant predictor of pentaBDE concentrations in handwipes and serum, explaining 16% of the variation of pentaBDE levels in handwipes and 20% of the variation of pentaBDE levels in serum. These results are consistent with the hypothesis that exposure to PBDEs via hands is a major contributor to body burden. Because we relied on participants to report hand-washing frequency, nondifferential misclassification in this measure is possible. Because hand washing had only two levels, we would expect nondifferential misclassification to bias our results toward the null.

Although hand washing was strongly associated with levels of pentaBDE congeners, it was not associated with BDE-183 and BDE-209 in handwipes (Table 2). One possible explanation is that the behavior of lower brominated penta congeners in the indoor environment may differ from that of higher brominated congeners such as BDE-183 and BDE-209. Using microscopy, researchers found evidence that BDE-209–containing particles in dust can be released from sources via weathering or abrasion, rather than through volatilization (Webster et al. 2009). If BDE-209 is generally attached to larger particles compared with pentaBDE congeners, they may be less likely to stick to the surface of hands, especially for long periods of time. As a result, the mass of BDE-209 (and possibly BDE-183) measured in handwipes would be less influenced by hand washing.

Although our results are consistent with multiple possible exposure pathways, the most probable is exposure to contaminated dust. PBDEs in dust may attach to hands via direct contact with surfaces and can then enter the body via two different exposure routes: incidental ingestion via hand-to-mouth contact or dermal absorption. We are unable to distinguish between these two possible exposure routes in this study. Another possibility is that vapor-phase PBDEs in indoor air absorb to skin, entering the body through dermal absorption (Weschler and Nazaroff 2008). However, the significant association between hand washing and pentaBDE concentrations in serum suggests that this is a less important pathway. Additional research is needed to distinguish between these possibilities.

Because lipids are soluble in isopropyl alcohol (the solvent used to collect handwipes), it is possible that handwipes measure PBDEs carried to the surface by lipids secreted by the skin rather than measuring exposure from the surrounding environment. In this reverse-causation scenario, PBDEs in handwipes correlate with PBDEs in serum because both are a measure of internal body burden. Although we are unable to rule out this reverse pathway, it appears unlikely because of the correlation between PBDEs in handwipes and office dust.

*Comparison with other studies.* Concentrations of PBDEs in office dust reported in this study exceed those seen in Japan (Suzuki et al. 2006), China (Huang et al. 2010), Belgium (Roosens et al. 2010), and Australia (Toms et al. 2009). pentaBDE concentrations in our office dust samples were also higher than concentrations reported in the United Kingdom, although BDE-209 concentrations were slightly lower (Harrad et al. 2008). Median concentrations of BDE-47, BDE-99, and BDE-100 in dust from offices in Michigan (USA) reported by Batterman et al. (2010) were slightly higher than median concentrations of the same congeners measured in the present study. Differences between reported concentrations in the United States and those reported in other countries are consistent with the use of penta mainly in the United States and deca worldwide. However, sample collection methods varied greatly among these studies, limiting our ability to make appropriate comparisons.

Measurements of most PBDE congeners in handwipes were lower than those previously reported in our handwipe pilot study (Stapleton et al. 2008), although BDE-138 and BDE-183 were higher. Higher BDE-183 measurements in handwipes from the present study may reflect past use of the octa formulation in office equipment.

GMs of individual pentaBDE congeners in serum from our study are 8–50% lower than those reported in National Health and Nutrition Examination Survey (NHANES) for 2003/2004 (Sjödin et al. 2008). This apparent difference in pentaBDE body burden could be due to phase-out of the penta formulation in consumer products or a result of our small sample size. BDE-209 has not been reported in NHANES, but a recent study of 24 adults also conducted in Boston, Massachusetts, detected BDE-209 in 8% of samples (< LOD to 6 ng/g lipid) (Johnson et al. 2010).

*Limitations.* Our small sample size limited the number of variables we could evaluate simultaneously in regression models and reduced our power to detect statistically significant associations. Because our population was not a random sample—in particular, everyone worked in an office—we cannot be certain that the associations reported here reflect the general population. For example, participants in our study were predominantly white, well-educated women from the Boston area who may wash their hands more or less frequently than others, or have cleaner or dirtier offices. Nevertheless, this may limit the generalizability but not the internal validity of our study.

We collected one office dust, handwipe, and serum sample per participant, at one point in time. This cross-sectional study design has different implications depending on sample type. Allen et al. (2008) found that concentrations of pentaBDE congeners in residential dust were strongly correlated over an 8-month period, and we would expect office dust concentrations to be similarly stable. PentaBDE congeners are estimated to have biological half-lives on the order of years (Geyer et al. 2004), so serum concentrations of these compounds should reflect long-term exposure. In contrast, handwipe samples presumably reflect recent exposure to the surrounding environment. Multiple handwipe samples collected in different microenvironments may better characterize total exposure via this pathway.

We detected BDE-209 in only 20% of serum samples, limiting the analyses we were able to perform with these data. The relatively short half-life of BDE-209 in serum may partially explain the low detection rates for this congener. Larger sample sizes or lower LODs would have allowed us to better characterize associations among BDE-209 in dust, handwipes, and serum.

## Conclusion

Our research suggests that exposure to PBDEs in the work environment may contribute to PBDE body burden for office workers. Associations between PBDEs found in office dust, handwipes, and serum suggest that potential exposure pathways may involve PBDEs on hands, either through incidental ingestion or dermal absorption. Hand washing may be a useful tool in decreasing exposure to chemicals in the indoor environment.

## Supplemental Material

(268 KB) PDF
