# Supplementary material for: Exposure to PBDEs in the Office Environment: Evaluating the Relationships Between Dust, Handwipes, and Serum
Source: Environ Health Perspect. 2011 Jun 30;119(9):1247–52. doi: 10.1289/ehp.1003271 (PMC3230398; doi:10.1289/ehp.1003271)
Supplement: (268 KB) PDF [file ehp.1003271.s001.pdf]

Supplemental Material

Exposure to PBDEs in the Office Environment: Evaluating the Relationship Between Dust, Handwipes and Serum

Deborah J. Watkins, Michael D. McClean, Alicia J. Fraser, Janice Weinberg, Heather M. Stapleton, Andreas Sjödin, Thomas F. Webster

Contents:

I. Supplemental Material, Text: Spearman Correlations within Sample Type 2

II. Supplemental Material, Table 1. Correlations of PBDE congeners in office dust 3

III. Supplemental Material, Table 2. Correlations of PBDE congeners in handwipes 5

IV. Supplemental Material, Figure 1. Scatter plot of PBDEs in office dust vs. handwipes 7

V. Supplemental Material, Table 3. Correlations of PBDE congeners in serum 8

VI. Supplemental Material, Figure 2. Conceptual Model of Exposure 9

References 10

Supplemental Material, Text: Spearman Correlations within Sample Type

Individual PBDE congeners in handwipes were correlated in a pattern reflecting the three commercial mixtures: Penta, Octa, and Deca. Penta congeners were all highly correlated with one another as well as with the sum Penta measure ($p<0.0001$ for all comparisons). BDE 183 in handwipes was moderately correlated with the Penta sum measure ($r=0.45$, $p=0.01$) but BDE 209 was not ($r=0.16$, $p=0.39$). Individual PBDE congeners measured in office dust were also correlated with one another in a pattern that generally represents the three commercial PBDE mixtures. However, BDE 183 and BDE 209 were more highly correlated than expected ($r=0.61$, $p=0.0003$) based on earlier work in homes (Allen et al. 2008), suggesting a possible common source of Octa and Deca. As Octa and Deca were commonly used in acrylonitrile-butadiene-styrene (ABS) and high-impact polystyrene (HIPS) polymers, respectively, for the housings of office equipment (Birnbaum and Cohen Hubal 2006), they may often occur together in the office environment. Both BDE 183 and BDE 209 were also moderately correlated with the sum PentaBDE measurement ($r=0.46$, $p=0.009$ and $r=0.45$, $p=0.01$ respectively). Principal component analysis confirmed that PBDE congeners in both handwipes and office dust clustered by commercial mixture, consistent with our previous findings (Allen et al. 2008).

Supplemental Material, Table 1. Correlations of PBDE congeners in office dust

	**BDE28/33**	**BDE47**	**BDE49**	**BDE66**	**BDE75**	**BDE85/155**	**BDE99**	**BDE100**	**BDE138**	**BDE153**
**BDE28/33**	1.00	0.80343	0.50010	0.78527	0.29091	0.68860	0.75782	0.77639	0.54208	0.60969
p-value		*<.0001*	*0.0042*	*<.0001*	*0.1124*	*<.0001*	*<.0001*	*<.0001*	*0.0016*	*0.0003*
**BDE47**		1.00	0.70202	0.73952	0.30616	0.80605	0.87137	0.95202	0.68508	0.71411
p-value			*<.0001*	*<.0001*	*0.0939*	*<.0001*	*<.0001*	*<.0001*	*<.0001*	*<.0001*
**BDE49**			1.00	0.53589	0.30010	0.54234	0.57944	0.64073	0.53992	0.53145
p-value				*0.0019*	*0.1009*	*0.0016*	*0.0006*	*0.0001*	*0.0017*	*0.0021*
**BDE66**				1.00	0.39072	0.66935	0.71089	0.76331	0.58145	0.59637
p-value					*0.0298*	*<.0001*	*<.0001*	*<.0001*	*0.0006*	*0.0004*
**BDE75**					1.00	0.47972	0.40383	0.32936	0.39617	0.39132
p-value						*0.0063*	*0.0243*	*0.0704*	*0.0274*	*0.0295*
**BDE85/155**						1.00	0.94395	0.89153	0.90000	0.86935
p-value							*<.0001*	*<.0001*	*<.0001*	*<.0001*
**BDE99**							1.00	0.95565	0.84153	0.86855
p-value								*<.0001*	*<.0001*	*<.0001*
**BDE100**								1.00	0.79597	0.82177
p-value									*<.0001*	*<.0001*
**BDE138**									1.00	0.97016
p-value										*<.0001*
**BDE153**										1.00

	**BDE154**	**BDE183**	**BDE196**	**BDE197**	**BDE201**	**BDE206**	**BDE207**	**BDE208**	**BDE209**	**ΣPentaBDE[a]**
**BDE28/33**	0.57154	0.37457	0.17740	0.16408	0.25227	0.19193	0.21756	0.22624	0.29001	0.81675
p-value	*0.0008*	*0.0379*	*0.3397*	*0.3778*	*0.1710*	*0.3010*	*0.2397*	*0.2210*	*0.1135*	*<.0001*
**BDE47**	0.68266	0.36653	0.28750	0.19597	0.31694	0.34194	0.35282	0.34758	0.37339	0.93992
p-value	*<.0001*	*0.0426*	*0.1168*	*0.2907*	*0.0824*	*0.0597*	*0.0516*	*0.0554*	*0.0385*	*<.0001*
**BDE49**	0.43871	0.31089	0.20403	0.10363	0.20645	0.38790	0.36855	0.35484	0.38226	0.63750
p-value	*0.0136*	*0.0887*	*0.2709*	*0.5791*	*0.2652*	*0.0311*	*0.0413*	*0.0501*	*0.0338*	*0.0001*
**BDE66**	0.58145	0.26290	0.09637	0.07097	0.15363	0.10968	0.13306	0.09274	0.18710	0.74718
p-value	*0.0006*	*0.1530*	*0.6060*	*0.7044*	*0.4093*	*0.5570*	*0.4755*	*0.6197*	*0.3135*	*<.0001*
**BDE75**	0.33764	0.15943	0.02926	0.03027	0.08254	0.01574	-0.03754	-0.06741	0.0121	0.36690
p-value	*0.0632*	*0.3916*	*0.8758*	*0.8716*	*0.6589*	*0.9330*	*0.8411*	*0.7186*	*0.9948*	*0.0423*
**BDE85/155**	0.85121	0.41089	0.30524	0.17540	0.37903	0.36008	0.34960	0.36855	0.39879	0.92177
p-value	*<.0001*	*0.0217*	*0.0950*	*0.3453*	*0.0355*	*0.0466*	*0.0539*	*0.0413*	*0.0263*	*<.0001*
**BDE99**	0.84758	0.46048	0.37984	0.24274	0.44274	0.43629	0.41411	0.42218	0.45242	0.97661
p-value	*<.0001*	*0.009*	*0.0351*	*0.1883*	*0.0126*	*0.0141*	*0.0206*	*0.0180*	*0.0106*	*<.0001*

**Table S1.** Correlations of PBDE congeners in office dust (cont.)										
	**BDE154**	**BDE183**	**BDE196**	**BDE197**	**BDE201**	**BDE206**	**BDE207**	**BDE208**	**BDE209**	**ΣPentaBDE[a]**
**BDE100**	0.79355	0.45968	0.36734	0.27540	0.41976	0.40726	0.40565	0.39677	0.43105	0.98145
p-value	*<.0001*	*0.0093*	*0.0421*	*0.1337*	*0.0187*	*0.0230*	*0.0236*	*0.0271*	*0.0155*	*<.0001*
**BDE138**	0.86411	0.59597	0.49315	0.35202	0.51169	0.47218	0.47500	0.46532	0.49113	0.81532
p-value	*<.0001*	*0.0004*	*0.0048*	*0.0521*	*0.0033*	*0.0073*	*0.0069*	*0.0083*	*0.0050*	*<.0001*
**BDE153**	0.88750	0.67782	0.57984	0.44194	0.58306	0.48226	0.50202	0.47661	0.51008	0.84556
p-value	*<.0001*	*<.0001*	*0.0006*	*0.0128*	*0.0006*	*0.0060*	*0.0040*	0.0067	*0.0034*	*<.0001*
	**BDE154**	**BDE183**	**BDE196**	**BDE197**	**BDE201**	**BDE206**	**BDE207**	**BDE208**	**BDE209**	**ΣPentaBDE[a]**
**BDE154**	1.00	0.48508	0.48589	0.27702	0.44556	0.41492	0.44274	0.42984	0.47097	0.80806
p-value		*0.0057*	*0.0056*	*0.1314*	*0.0120*	*0.0203*	*0.0126*	*0.0158*	*0.0075*	*<.0001*
**BDE183**		1.00	0.82782	0.92621	0.76613	0.57944	0.64435	0.57097	0.60847	0.46008
p-value			*<.0001*	*<.0001*	*<.0001*	*0.0006*	*<.0001*	*0.0008*	*0.0003*	*0.0092*
**BDE196**			1.00	0.83911	0.82419	0.70806	0.76613	0.68065	0.71048	0.55806
p-value				*<.0001*	*<.0001*	*<.0001*	*<.0001*	*<.0001*	*<.0001*	*0.0011*
**BDE197**				1.00	0.76331	0.54677	0.61694	0.52661	0.56774	0.32298
p-value					*<.0001*	*0.0015*	*0.0002*	*0.0023*	*0.0009*	*0.0764*
**BDE201**					1.00	0.77782	0.83629	0.81290	0.79597	0.26411
p-value						*<.0001*	*<.0001*	*<.0001*	*<.0001*	*0.1511*
**BDE206**						1.00	0.95968	0.95202	0.96978	0.42056
p-value							*<.0001*	*<.0001*	*<.0001*	*0.0185*
**BDE207**							1.00	0.97984	0.97218	0.41331
p-value								*<.0001*	*<.0001*	*0.0208*
**BDE208**								1.00	0.96250	0.41169
p-value									*<.0001*	*0.0214*
**BDE209**									1.00	0.44960
p-value										*0.0112*
**ΣPentaBDE[a]**										1.00

[a]ΣPentaBDE comprises BDE28/33, 47, 99, 100, and 153 (congeners detected in >50% of samples within all three media: dust, handwipes, and serum).

Supplemental Material, Table 2**.** Correlations of PBDE congeners in handwipes

	**BDE28/33**	**BDE47**	**BDE49**	**BDE66**	**BDE75**	**BDE85/155**	**BDE99**	**BDE100**	**BDE138**
**BDE28/33**	1.00	0.77022	0.72254	0.77362	0.41329	0.61467	0.72019	0.73008	0.66569
p-value		*<.0001*	*<.0001*	*<.0001*	*0.0208*	*0.0002*	*<.0001*	*<.0001*	*<.0001*
**BDE47**		1.00	0.82482	0.85152	0.58323	0.80851	0.93427	0.95323	0.88435
p-value			*<.0001*	*<.0001*	*0.0006*	*<.0001*	*<.0001*	*<.0001*	*<.0001*
**BDE49**			1.00	0.90741	0.55525	0.69517	0.81514	0.82220	0.76785
p-value				*<.0001*	*0.0012*	*<.0001*	*<.0001*	*<.0001*	*<.0001*
**BDE66**				1.00	0.52800	0.77772	0.88420	0.88541	0.83006
p-value					*0.0023*	*<.0001*	*<.0001*	*<.0001*	*<.0001*
**BDE75**					1.00	0.59869	0.70849	0.67571	0.71821
p-value						*0.0004*	*<.0001*	*<.0001*	*<.0001*
**BDE85/155**						1.00	0.89624	0.88918	0.85099
p-value							*<.0001*	*<.0001*	*<.0001*
**BDE99**							1.00	0.99476	0.95970
p-value								*<.0001*	*<.0001*
**BDE100**								1.00	0.95444
p-value									*<.0001*
**BDE138**									1.00
	**BDE153**	**BDE154**	**BDE183**	**BDE197**	**BDE207**	**BDE208**	**BDE209**	**ΣPentaBDE[a]**	
**BDE28/33**	0.67736	0.69971	0.47983	0.30411	0.01928	-0.07048	0.05629	0.75368	
p-value	*<.0001*	*<.0001*	*0.0063*	*0.0963*	*0.9180*	*0.7064*	*0.7636*	*<.0001*	
**BDE47**	0.90583	0.91395	0.45832	0.21118	0.11059	0.00899	0.12844	0.97702	
p-value	*<.0001*	*<.0001*	*0.0095*	*0.2541*	*0.5537*	*0.9617*	*0.4911*	*<.0001*	
**BDE49**	0.79279	0.79754	0.34267	0.20432	-0.07673	-0.15965	-0.10879	0.83169	
p-value	*<.0001*	*<.0001*	*0.0591*	*0.2702*	*0.6816*	*0.3909*	*0.5602*	*<.0001*	
**BDE66**	0.86007	0.86202	0.40138	0.22138	-0.05596	-0.14438	-0.02118	0.87291	
p-value	*<.0001*	*<.0001*	*0.0252*	*0.2314*	*0.7650*	*0.4384*	*0.9099*	*<.0001*	
**BDE75**	0.70327	0.69350	0.32901	0.28711	0.26363	0.15859	0.38140	0.66883	
p-value	*<.0001*	*<.0001*	*0.0707*	*0.1173*	*0.1519*	*0.3942*	*0.0343*	*<.0001*	
**BDE85/155**	0.87837	0.88426	0.30443	0.11783	0.02430	0.10804	0.12555	0.86942	
p-value	*<.0001*	*<.0001*	*0.0959*	*0.5278*	*0.8967*	*0.5629*	*0.5009*	*<.0001*	
**BDE99**	0.99072	0.99082	0.44943	0.25081	0.09006	-0.00300	0.19236	0.98387	
p-value	*<.0001*	*<.0001*	*0.0112*	*0.1735*	*0.6299*	*0.9872*	*0.2999*	*<.0001*	
**BDE100**	0.98185	0.98315	0.45347	0.23988	0.08336	-0.00621	0.15687	0.99153	
p-value	*<.0001*	*<.0001*	*0.0104*	*0.1937*	*0.6557*	*0.9736*	*0.3994*	*<.0001*	

**Table S2.** Correlations of PBDE congeners in handwipes (cont.)									
	**BDE153**	**BDE154**	**BDE183**	**BDE197**	**BDE207**	**BDE208**	**BDE209**	**ΣPentaBDE[a]**	
**BDE138**	0.96294	0.95815	0.38869	0.17742	0.05976	-0.04355	0.18232	0.94249	
p-value	*<.0001*	*<.0001*	*0.0307*	*0.3397*	*0.7495*	*0.8160*	*0.3263*	*<.0001*	
**BDE153**	1.00	0.99627	0.45063	0.27903	0.08170	-0.02559	0.17868	0.96653	
p-value		*<.0001*	*0.0110*	*0.1285*	*0.6622*	*0.8913*	*0.3362*	*<.0001*	
**BDE154**		1.00	0.43721	0.26234	0.05973	-0.04520	0.17322	0.96863	
p-value			*0.0139*	*0.1539*	*0.7496*	*0.8092*	*0.3514*	*<.0001*	
**BDE183**			1.00	0.58038	0.64386	0.45749	0.38834	0.45186	
p-value				*0.0006*	*<.0001*	*0.0097*	*0.0309*	*0.0107*	
**BDE197**				1.00	0.40807	0.26564	0.24098	0.24609	
p-value					*0.0227*	*0.1487*	*0.1916*	*0.1820*	
**BDE207**					1.00	0.88315	0.59111	0.10095	
p-value						*<.0001*	*0.0005*	*0.5889*	
**BDE208**						1.00	0.44812	0.01135	
p-value							*0.0115*	*0.9517*	
**BDE209**							1.00	0.15828	
p-value								*0.3951*	
**ΣPentaBDE[a]**								1.00	

[a]ΣPentaBDE comprises BDE28/33, 47, 99, 100, and 153 (congeners detected in >50% of samples within all three media: dust, handwipes, and serum)

Supplemental Material, Figure 1. Scatter plot of PBDEs in office dust vs. handwipes (n=31)

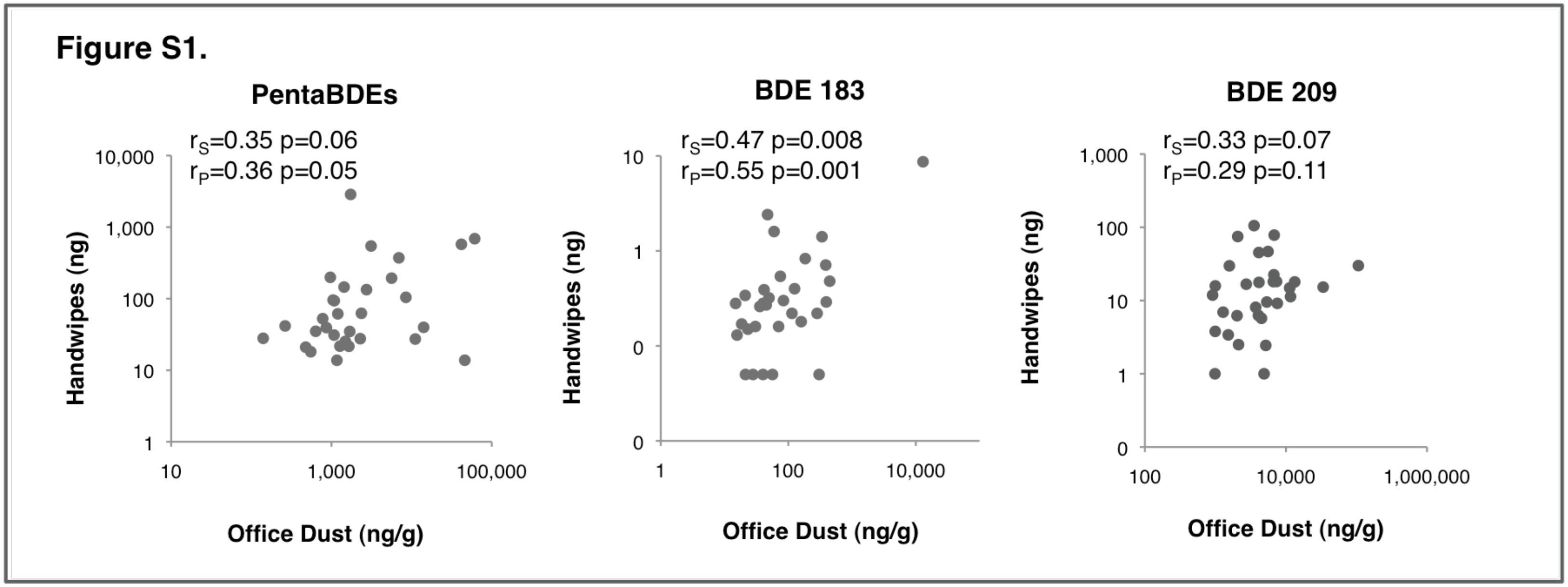

r_S =Spearman Correlation Coefficient; r_P =Pearson Correlation Coefficient of natural log-transformed data

Supplemental Material, Table 3. Correlations of PBDE congeners in serum

	BDE28	BDE47	BDE99	BDE100	BDE153	BDE209	ΣPentaBDE[a]
**BDE28**	1.00	0.89412	0.75485	0.83517	0.75945	-0.05595	0.87614
p-value		*<.0001*	*<.0001*	*<.0001*	*<.0001*	*0.7690*	*<.0001*
**BDE47**		1.00	0.91340	0.96863	0.73807	-0.03834	0.96729
p-value			*<.0001*	*<.0001*	*<.0001*	*0.8406*	*<.0001*
**BDE99**			1.00	0.94365	0.76222	-0.02376	0.92563
p-value				*<.0001*	*<.0001*	*0.9008*	*<.0001*
**BDE100**				1.00	0.80303	0.02755	0.97529
p-value					*<.0001*	*0.8851*	*<.0001*
**BDE153**					1.00	-0.02308	0.85562
p-value						*0.9036*	*<.0001*
**BDE209**						1.00	-0.02074
p-value							*0.9134*
**ΣPentaBDE[a]**							1.00

[a]ΣPentaBDE comprises BDE28/33, 47, 99, 100, and 153 (congeners detected in >50% of samples within all three media: dust, handwipes, and serum)

Supplemental Material, Figure 2. Conceptual Model of Exposure

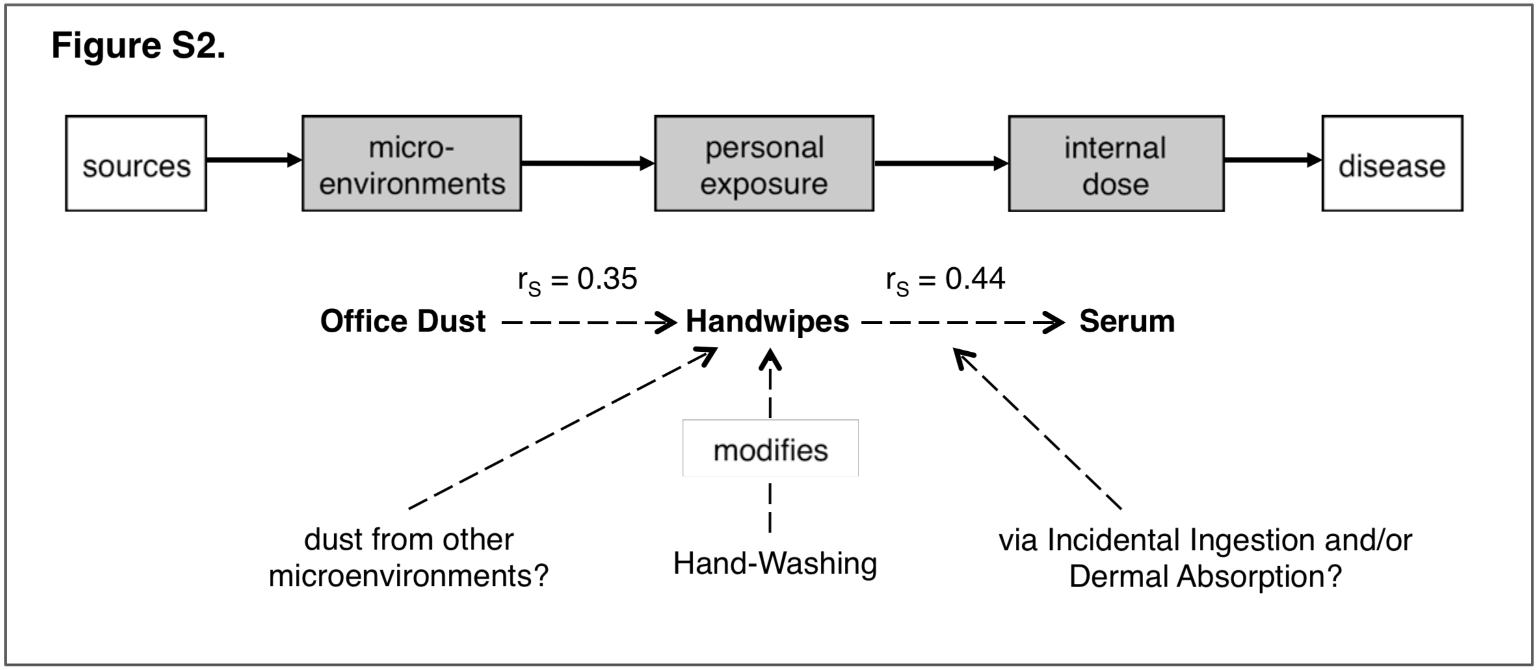

Office dust is a measure of PBDEs in the microenvironment, handwipes a measure of personal exposure, and serum a measure of internal dose. r_S =Spearman Correlation Coefficient

References

Allen JG, McClean MD, Stapleton HM, Webster TF. 2008. Critical factors in assessing exposure to PBDEs via house dust. Environ Int 34(8):1085-1091.

Birnbaum LS, Cohen Hubal EA. 2006. Polybrominated diphenyl ethers: A case study for using biomonitoring data to address risk assessment questions. Environ Health Perspect 114(11):1770-1775.
